# Live imaging of Rab8 trafficking defects in *cc2d2a* mutant zebrafish

**DOI:** 10.1186/2046-2530-1-S1-O29

**Published:** 2012-11-16

**Authors:** R Bachmann-Gagescu, IG Phelps, A Forbes, D Doherty, CB Moens

**Affiliations:** 1 University of Washington, USA; 2 Fred Hutchinson Cancer Research Center, USA

## 

Primary cilia provide a means of regulating and concentrating receptors and other proteins essential for transmission of sensory signals or signaling pathways. Consequently, a thorough understanding of the sorting and trafficking mechanisms that allow appropriate protein delivery to the ciliary compartment is crucial to our understanding of ciliary function in development, homeostasis and disease. Recent work has demonstrated that ciliopathy proteins, such as CC2D2A, are localized to the transition zone at the base of the cilium and function to regulate protein entry into the ciliary compartment. Upstream of the cilium, small GTPases from the Rab family participate in controlling trafficking of ciliary-directed proteins. Here, we describe the relationship between the transition zone protein Cc2d2a and Rab8 trafficking in photoreceptors. Detailed phenotypic analysis of a zebrafish *cc2d2a* mutant reveals typical ciliopathy phenotypes including cystic kidneys and abnormal photoreceptor outer segments. Mislocalization of opsins in mutant photoreceptors and massive accumulation of vesicles suggest a role for Cc2d2a in vesicle trafficking. Partial *rab8* knockdown enhances the photoreceptor phenotype of *cc2d2a* mutants. A fluorescently-tagged Rab8 transgene demonstrates a punctate localization pattern for Rab8 in photoreceptors that is disrupted in the absence of Cc2d2a. Live imaging of Rab8 trafficking in zebrafish photoreceptors reveals complex and highly dynamic movements of Rab8 puncta towards different cellular destinations, including the outer segment and the synapse, which are differentially affected by loss of Cc2d2a function.

